# Mitochondrial dysfunction and alcohol-associated liver disease: a novel pathway and therapeutic target

**DOI:** 10.1038/s41392-020-0128-8

**Published:** 2020-03-03

**Authors:** Mohamed A. Abdallah, Ashwani K. Singal

**Affiliations:** 1 Department of Medicine, University of South Dakota, Sanford School of Medicine, Sioux falls, SD USA; 2 Division of Gastroenterology and Hepatology, University of South Dakota, Sanford School of Medicine, Sioux falls, SD USA; 3 Avera University Hospital Transplant Institute & Chief Clinical Research Affairs, Transplant Hepatology, Institute of Human Genetics Research, Sioux Falls, SD 57105 USA

**Keywords:** Molecular biology, Gastroenterology, Molecular biology, Gastroenterology, Molecular biology

In a recent study in *signal transduction and targeted therapy*, Zhou et al. provided novel experimental data on the role of DNA-dependent protein kinase catalytic subunit (DNA-PKcs) in causing abnormalities in the structure and function of mitochondria of hepatocytes, leading to liver injury and alcohol-associated liver disease (ALD).^1^

Mitochondria play a crucial role in cellular energy metabolism and in reactive species formation.^2^ Several studies in both animal and human models have demonstrated that alcohol intake alters mitochondrial morphology and function by causing impairment of mitochondrial biogenesis, mitochondrial DNA damage, lipid accumulation, oxidative stress, and hepatocellular apoptosis.^2,3^ Therefore, identification of the mechanisms through which alcohol induces mitochondrial dysfunction and liver injury is vital for understanding of the pathogenesis of ALD.

Mitochondrial fission and mitophagy are important processes for maintaining mitochondrial homeostasis.^4^ In a study conducted by the same group, impairment of mitochondrial fission and mitophagy through upregulation of nuclear receptor subfamily 4 group A member 1 (NR4A1) and subsequent activation of the DNA-PKcs/p53 pathway resulted in mitochondrial dysfunction, with reduction in mitochondrial potential, oxidative stress, calcium overload, mitochondrial respiratory collapse, and ATP deficiency. NR4A1 gene encodes the steroid–thyroid hormone–retinoid receptor superfamily, and the encoded protein acts as transcription factor. Genetic deletion of NR4A1 or DNA-PKcs reversed mitochondrial dysfunction and prevented diet-induced nonalcoholic fatty liver disease.^5^ The role of NR4A1/DNA-PKcs/P53 pathway in the regulation of mitochondrial fission and mitophagy, however, has not been studied in ALD.

In this issue of *signal transduction and targeted therapy*, Zhou et al. demonstrate the role of DNA-PKcs in the pathogenesis of ALD. In ethanol-fed mice, DNA-PKcs was significantly upregulated and activated in comparison with controls. The activation of DNA-PKcs was associated with the development of severe ALD in wild-type (WT) mice treated with alcohol, with hepatocyte vacuolation, fibrosis, steatosis, increased lipid droplets, and elevation in serum alanine aminotransferase (ALT), aspartate aminotransferase (AST), and AST/ALT ratio. These findings were absent in liver-specific DNA-PKcs knockout (DNA-PKcs*LKO*) mice treated with alcohol. In addition, transcription of inflammation/fibrosis markers, such as monocyte chemotactic protein 1, macrophage inflammatory protein 1α, interleukin 8, and increased mitochondrial apoptosis-related proteins were seen in WT mice treated with alcohol, but not in DNA-PKcs knockout mice treated with alcohol. Other markers for mitochondrial dysfunction, such as oxidative stress, impaired mitochondrial biogenesis, and distorted morphology were also detected in alcohol-treated WT mice but not in DNA-PKcs knockout mice.

Alcohol-induced mitochondrial fission characterized by formation of small, round fragmented mitochondria was mediated by DNA-PKcs upregulation, with subsequent activation of dynamin-related protein 1(Drp1)-related mitochondrial fission and genomic instability. On the other hand, impairment of mitophagy induced by alcohol was closely associated with reduced ATP and defective mitochondrial–lysosomes fusion in hepatocytes. FUNDC1, a novel receptor that activates mitophagy via dephosphorylation at Ser13 (ref. ^6^), can be deactivated by phosphorylation induced by alcohol. Also, PINK 1 and Parkin, mitophagy-related markers, are downregulated by alcohol.^4^ These alcohol-induced changes in mitophagy were reversed to normal with deletion of DNA-PKcs. In mice with DNA-PKcs deletion, silencing of FUNDC1 resulted in mitophagy impairment.

**Fig. 1:**
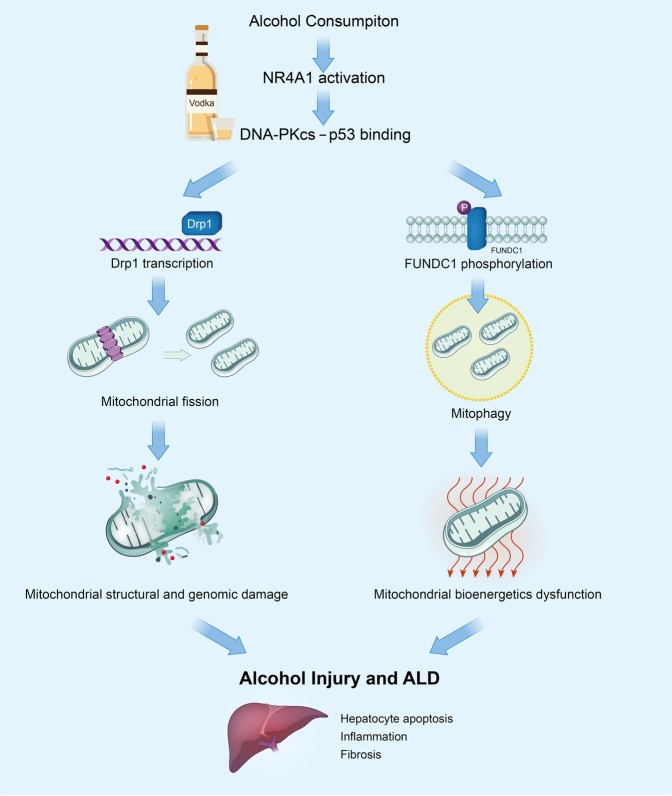
DNA dependent protein kinase and mitochondrial dysfunction in alcohol-associated liver disease. Alcohol-induced upregulation of NR4A1 with subsequent DNA-PKcs and p53 bond resulting in **a** Drp1 activation, resulting in mitochondrial structural damage and **b** inhibition of FUNDC1-mediated protective mitophagy, resulting in mitochondrial bioenergetics defect. These mitochondrial abnormalities result in hepatocyte apoptosis, inflammation, and fibrosis with the development of alcohol-associated liver disease

The investigators also showed that the DNA-PKcs regulation of mitochondrial fission and mitophagy was mediated through P53. Alcohol-induced phosphorylation of p53 (the primary transcription promoter for Drp at Ser 15) through DKNA-PKcs resulted in increased Drp1 transcription, and subsequently Drp-1-mediated mitochondrial fission. In addition, P53-mediated FUNDC1 phosphorylation through the phosphorylation of casein kinase 2 (CK2) with the resultant inhibition of mitochondrial mitophagy (Fig. 1).

In response to DNA damage, DNA-PKcs plays a vital role in DNA double-strand break repair and genomic stability, through the interaction with Ku80 and the formation of DNA-PK (ref. ^7^). However, in prolonged insults, DNA-PKcs can be activated by orphan NR4A1, thereby leading to DNA-PKcs disassociation from Ku80 and potentially binding to p53 (ref. ^8^). This response to cellular injury by increasing NR4A1 can be mediated by alcohol. Zhou et al. demonstrated that binding of DNA-PKcs to p53 was NR4A1 dependent and that NR4A1 silencing resulted in the suppression of DNA-PKcs, p53, and reversal and suppression of both alcohol-induced mitochondrial fission and mitophagy. Moreover, NR4A1 knockout (NR4A1-KO) mice treated with alcohol for 16 weeks had less hepatocyte vacuolation, fibrosis, steatosis, and caspase-9-related mitochondrial apoptosis in comparison to the alcohol-treated WT.

Given the increasing morbidity and mortality associated with ALD worldwide,^9^ better understanding of the mechanisms of alcohol-induced liver injury is crucial. Research conducted by Zhou et al. highlights the role of NR4A1/DNA-PKcs/p53 pathway in mediating mitochondrial structural and functional abnormalities, resulting in liver injury induced by alcohol. Exploring other non-mitochondrial mechanisms of ALD pathogenesis is also needed, including but not limited to role of Patatin-like phospholipase domain containing protein 3 (*PNPLA3*) and other genetic polymorphisms, gut-liver axis and microbiota, hepatic regeneration, and inflammation with extracellular vesicles, as basis for developing newer pharmacological therapies for this disease.^10^ Translational prospective well-designed studies are suggested to validate the novel findings of this study in human phenotype of ALD as basis for identifying newer therapeutic targets for ALD.
